# Supplementary material for: Targets of SPEECHLESS and FAMA control guard cell division and expansion in the late stomatal lineage
Source: Development. 2026 Jun 10;153(11):dev205374. doi: 10.1242/dev.205374 (PMC13286351; doi:10.1242/dev.205374)
Supplement: Supplementary file 1 [file develop-153-205374_review_history.pdf]

Targets of SPEECHLESS and FAMA control guard cell division and expansion in the late stomatal lineage

Pablo González-Suárez, Yoëlle Hilbers, Alanta Budrys, Ao Liu, Sandra Richter, Dominique C. Bergmann and Margot E. Smit

DOI: 10.1242/dev.205374

Editor: Steve Wilson

Review timeline

Original submission:	17 November 2025
Editorial decision:	19 December 2025
First revision received:	24 March 2026
Editorial decision:	24 April 2026
Second revision received:	7 May 2026
Accepted:	8 May 2026

Original submission

First decision letter

MS ID#: dev.205374

MS TITLE: Targets of SPEECHLESS and FAMA control guard cell division and expansion in the late stomatal lineage

AUTHORS: Pablo González-Suárez, Yoëlle Hilbers, Alanta Budrys, Ao Liu, Sandra Richter, Dominique C. Bergmann and Margot Smit

Dear Margot,

I have now received all the referees' reports on the above manuscript, and have reached a decision. The referees' comments are appended below.

As you will see, the referees express considerable interest in your work, but have some significant criticisms and suggestions to improve your manuscript. Reviewer 2 in particular would like additional experimental data to ensure that the conclusions you draw from the study are robust. If you are able to revise the manuscript along the lines suggested, I will be happy receive a revised version of the manuscript. Please also note that Development will normally permit only one round of major revision. If it would be helpful, you are welcome to contact us to discuss your revision in greater detail. Please send us a point-by-point response indicating your plans for addressing the referees' comments, and we will look over this and provide further guidance.

Please attend to all of the reviewers' comments and ensure that you upload both a 'clean' version of your Word file, along with a highlighted version clearly showing where you have made changes in the revised manuscript. Please avoid using 'Tracked changes' in Word files as these are lost in PDF conversion. I should be grateful if you would also provide a point-by-point response detailing how you have dealt with the points raised by the reviewers in the 'Response to Reviewers' box. If you do not agree with any of their criticisms or suggestions please explain clearly why this is so.

Reviewer 1

© 2026. Published by The Company of Biologists under the terms of the Creative Commons Attribution License (https://creativecommons.org/licenses/by/4.0/).

Advance summary and potential significance to field

This manuscript demonstrates that the relative levels and activities of the transcription factors SPCH and FAMA in the later stages of stomatal differentiation strictly regulate cell division and morphology. Notably, the study challenges the classical view that the functions of these two factors are strictly temporally separated. The authors show that SPCH is expressed in the late stomatal lineage and that its overexpression can induce symmetric cell division. This suggests that SPCH function is not restricted to asymmetric division but is dependent on cell identity. Furthermore, the authors identified novel downstream target genes for both factors, demonstrating that the precise spatiotemporal regulation of these targets is essential for the proper size, shape, and function of guard cells.
I commend the authors for the extensive generation of transformants and the detailed observation and analysis presented. However, I have several concerns, ranging from major conceptual points to minor formatting issues, which are listed below.

Comments for the author

Major Comments
1. To analyze SPCH function in the late stomatal lineage, the authors employed an ectopic expression system. However, phenotypes resulting from overexpression can be difficult to interpret due to potential artifacts. Loss-of-function experiments are generally considered the most direct approach to elucidating gene function. Did the authors attempt to perform SPCH knockdown (or conditional knockout) experiments specifically in the late stomatal lineage?

2. Line 141: To what extent do SPCH- and FAMA-expressing cells overlap when observed with fluorescent reporters? Is co-expression limited to a small fraction of cells, or is it widespread? Quantitative data are required to clarify this point. Furthermore, does the degree of co-expression align with the scRNA-seq data? If there is a discrepancy, please discuss the potential causes. On a related note, SPCH expression has been analyzed in previous studies; why was this late-stage expression overlooked in the past?

3. Lines 255-267: The rationale for altering the DEG analysis method based solely on GO analysis results, without apparent scrutiny of the specific gene lists, is unclear. Additionally, the description of the subsequent analysis is too brief in the Results section compared to the explanation of why the change was made. Please provide a more detailed explanation of the analytical process. Did the authors include data for DEX induction at 0 hours as a control?

4. Fig. 2: Quantitative results are necessary for this figure. Currently, only a few representative images are presented, making it difficult to assess the frequency of the observed phenomena. Providing quantitative data would significantly enhance the reliability of these findings.

5. It is known that the FAMA gene is expressed in myrosin cells in addition to guard cells. Is there a possibility that myrosin cells were included in the FAMA-positive population collected for expression analysis? At the very least, the authors should mention in the text that the FAMA promoter is also active in myrosin cells.

Minor points
6. Line 32: The background description in the Abstract is too broad. Focusing on specific issues relevant to this study would improve readability.
7. Line 54: Is the keyword "FMA" necessary?
8. Line 137: Should the citation be "Zhang et al., 2021"?
9. Line 143: Similar to Fig. 1, Fig. S2 requires calibration bars for fluorescence intensity and legends for the pseudocolors.
10. Line 210: The sentence starts with "Interestingly," but is the context clear to readers without a reference? Citing a study such as (Ohashi-Ito and Bergmann, 2006) might help explain why this result is notable.
11. Fig. 2: Please specify which panels correspond to the magnified views in C and J.
12. Fig. 2: The figure legend for panel C is duplicated.

© 2026. Published by The Company of Biologists under the terms of the Creative Commons Attribution License (https://creativecommons.org/licenses/by/4.0/).

13. Fig. S6: The figure panels and the legend do not match (e.g., there is no legend for panel F). What does "17%<10" in panel F represent? Please also explain "total, long, trans" in panel J. Does the number "8" indicate the sample size (n)?
14. Lines 223-225: It is unclear whether the authors are discussing the effects of ectopic overexpression or endogenous SPCH function. Please clarify.
15. Line 275: Should the reference be "Figures 3F-I" instead of "3F-G"?
16. Fig. S8A / Table S2: Since GLV1 is shown in Fig. S8A, it should also be listed in Table S2.
17. Fig. 4: Panels D and L are both labeled "C". In panel L, the data points for "FAMAp::WSB opening" are solid dots, whereas others are open circles; please ensure consistency.
18. Lines 372-374: The text description does not match the cited data in Fig. 5E (Nucleus area). The correct citation appears to be Fig. S11G-I.
19. Fig. 5F: I was unable to locate the reference to Fig. 5F in the main text. Please check if it is cited.

Reviewer 2

Advance summary and potential significance to field

This manuscript presents a compelling and timely investigation into the regulatory mechanisms controlling the late stages of stomatal development. The study effectively challenges the classical, linear model of bHLH transcription factor action by demonstrating that SPCH and FAMA are co-expressed and functionally interact in GMCs and young GCs. The combination of precise genetic manipulations, cell type-specific transcriptomics, and detailed phenotypic characterization provides strong evidence for a model where the relative levels and activities of SPCH and FAMA fine-tune the final steps of stomatal formation. Here are the major Concerns:
1. The data strongly suggest that SPCH and FAMA compete for binding to overlapping genomic targets. However, this competition model is primarily inferred from the similar phenotypes of the SRDX fusions and the rescue experiments in fama. More direct evidence is needed. Is there direct interaction of FAMA with SPCH? Do they enrich on the same promoter regions of their common target genes?
2. The results are descriptive in several key areas. For instance, the statement that SPCH-induced divisions are "mostly longitudinal" (Figure 1) requires rigorous quantification across multiple samples and time points. The percentages of different division types (longitudinal, transverse, aborted) should be quantified and statistically compared.
3. The finding that SRDX-FAMA can prevent tumor formation in the fama mutant but not rescue differentiation is fascinating. The authors interpret this as FAMA's differentiation function requiring its activator role. However, an alternative explanation is that the SRDX domain simply renders the protein non-functional, and the prevention of tumors is an artifact (e.g., by sequestering essential co-activators needed for division).
4. For determine the target genes of FAMA and SPCH in stomatal development, the authors used stomatal density, however, the density is often influenced by the size of pavement cells, therefore, the stomatal index is required to support their function.
5. In Figures 4C and S11, the authors measured the distance between guard cell membranes. It is important to note that plant cells possess a cell wall; therefore, are these measurements indicative of pore apertures?
6. It was reported that SCAP1 has a broader expression pattern in stomatal linage cells, therefore, it cannot be used a guard cell marker line.
7. In most figure legends, the descriptions are not clearly articulated.

Reviewer 3

Advance summary and potential significance to field

Summary
This manuscript addresses stomatal guard cell differentiation from the perspective of master transcription factor regulation. Building on a previously unexpected observation from the Bergmann lab that the transcription factor SPEECHLESS (SPCH) is expressed during late stages of the stomatal

© 2026. Published by The Company of Biologists under the terms of the Creative Commons Attribution License (https://creativecommons.org/licenses/by/4.0/).

lineage (Lopez-Anido et al. Developmental Cell), the authors perform a thorough and well-designed analysis of SPCH and its sister transcription factor FAMA.
In the first half of the paper, the authors compare inducible expression systems for SPCH and FAMA, as well as SRDX-fused chimeric repressor versions (SPCH-SRDX and FAMA-SRDX), to dissect functional differences between these transcription factors. In the second half, they carefully analyze a set of shared downstream genes of SPCH and FAMA using misexpression approaches, ultimately classifying their roles in guard cell division and expansion and successfully expanding the regulatory network.
Overall, this study provides important molecular insight into how guard cell maturation and morphogenesis are regulated, and I strongly recommend this manuscript for publication in Development. I do not think additional experiments are strictly required. However, clarification of several points and the inclusion of additional figures for data that are currently only described in the text would substantially improve clarity and accessibility for readers.

Major comments
1) Discussion of SPCH-FAMA interaction at the protein level
It would benefit the reader to include a discussion of whether SPCH and FAMA physically interact at the protein level. In the FAMA-TurboID dataset reported by Mair et al. (eLife, 2019), SPCH was not detected among FAMA interactors. It may be useful to explicitly mention this in the Discussion and comment on how this result fits with the current findings.

2) Definition and validation of "transcriptional targets" in the title and Results
The title refers to "transcriptional targets of SPEECHLESS and FAMA," but it is not always clear whether the genes analyzed in the second half of the manuscript are direct targets, or whether they are positively or negatively regulated by SPCH and/or FAMA.
2-1) Clarification of target selection strategy
Although I read the Methods section carefully, the logic used to narrow down candidates was not entirely clear. Specifically, it was difficult to understand whether the authors focused primarily on genes already known to be involved in stomatal maturation, or whether the candidate genes were selected purely based on intersection of multiple omics datasets.
It would be very helpful if the authors could clarify this point explicitly. For example, a schematic or Venn diagram illustrating how the candidate list was reduced (e.g. from ~195 genes to 23 genes) would greatly improve clarity.
2-2) Inclusion of omics data in the main figures
In addition, I strongly recommend that representative omics data supporting the analyzed gene set be shown in the main figures rather than only being described in the text. Specifically, the following would be very helpful:
* Screenshots of ChIP-seq peaks in IGV for representative target genes
* Expression plots (mock vs. dex) for FAMA, SPCH, FAMA-SRDX, and SPCH-SRDX induction lines
* Expression patterns of these genes projected onto the UMAP from the single-cell analysis by Lopez-Anido et al. (Developmental Cell)

These additions would substantially strengthen the link between the transcriptomic analyses and the functional experiments.

Minor comments
* Line 44: The wording may give the impression that a physical interaction between SPCH and FAMA was directly tested; this could be clarified.
* Line 68: The correct reference may be Ohashi-Ito and Bergmann, 2006.
* Line 394 / Figure 5F: Consider adding SCAP1 to the model diagram.
* Line 484: In the myrosin cell lineage, WSB represses SCAP1, whereas this does not appear to occur in the stomatal lineage. This contrast is very interesting and may deserve further discussion.

First revision

Author response to reviewers' comments

© 2026. Published by The Company of Biologists under the terms of the Creative Commons Attribution License (https://creativecommons.org/licenses/by/4.0/).

Reviewer 1
This manuscript demonstrates that the relative levels and activities of the transcription factors SPCH and FAMA in the later stages of stomatal differentiation strictly regulate cell division and morphology. Notably, the study challenges the classical view that the functions of these two factors are strictly temporally separated. The authors show that SPCH is expressed in the late stomatal lineage and that its overexpression can induce symmetric cell division. This suggests that SPCH function is not restricted to asymmetric division but is dependent on cell identity. Furthermore, the authors identified novel downstream target genes for both factors, demonstrating that the precise spatiotemporal regulation of these targets is essential for the proper size, shape, and function of guard cells. I commend the authors for the extensive generation of transformants and the detailed observation and analysis presented. However, I have several concerns, ranging from major conceptual points to minor formatting issues, which are listed below.

Major comments

1. To analyze SPCH function in the late stomatal lineage, the authors employed an ectopic expression system. However, phenotypes resulting from overexpression can be difficult to interpret due to potential artifacts. Loss-of-function experiments are generally considered the most direct approach to elucidating gene function. Did the authors attempt to perform SPCH knockdown (or conditional knockout) experiments specifically in the late stomatal lineage?

We agree with the reviewer that loss-of-function experiments are the most straight-forward to analyze and apologize that we did not make it clear that this work extends earlier loss-of-function experiments introduced in Lopez-Anido et al., 2021. Briefly, that paper showed that SPCHcds complementation has reduced SPCH in late stages, resulting in additional asymmetric cell divisions and loss of stomatal fate: cells diverted and gained pavement cell-like characteristics. Similar phenotypes were observed when SPCH levels were reduced in the late lineage using an artificial microRNA targeting SPCH driven by the *MUTE* promoter. These results had shown that late SPCH acts in stomatal fate maintenance and division program control in the late stomatal lineage and formed a starting point for this work. What remained a mystery from the early work is what specific targets of SPCH mediate this function in the late lineage, and this is what we addressed in this work. We have added a more explicit call out to the earlier loss-of-function experiments in lines 88-90 and 152-154 to provide more detail. In the early stages of this project, we also generated a FAMAp::SPCHamiR line and observed some single guard cells (GCs), consistent with SPCH having a role in promoting GC division, a phenotype consistent with our data and conclusions in this paper. The phenotypes were of low penetrance in the amiRNA lines, however, and we thought our other experimental data were stronger, so we did not include this in the manuscript. In the images below we show examples of the phenotype.

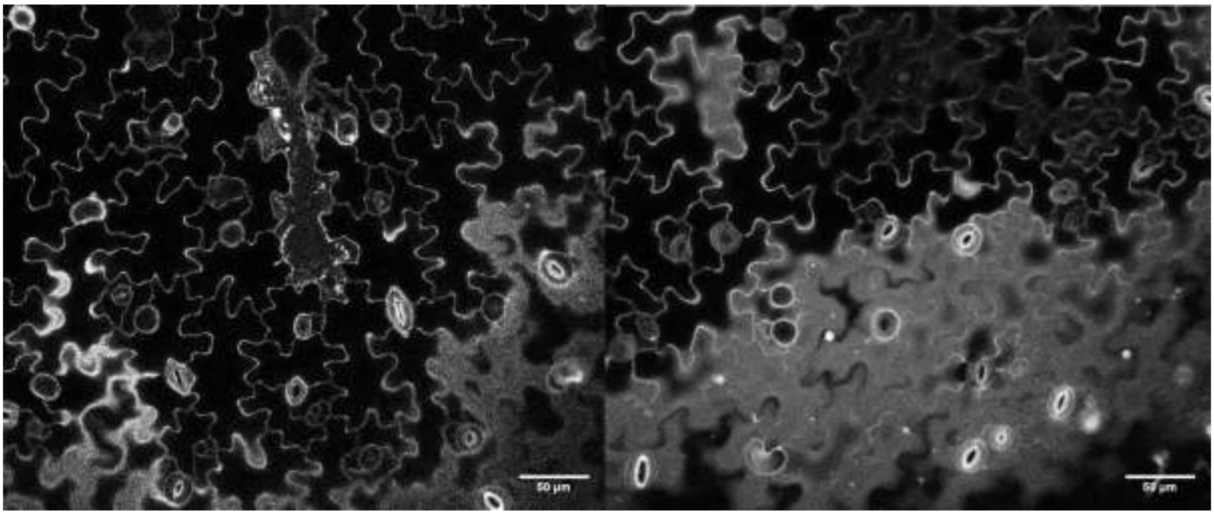

The leaf epidermis of a FAMAp::SPCHamiR shows large single Guard Cells 6dag.

© 2026. Published by The Company of Biologists under the terms of the Creative Commons Attribution License (https://creativecommons.org/licenses/by/4.0/).

2. Line 141: To what extent do SPCH- and FAMA-expressing cells overlap when observed with fluorescent reporters? Is co-expression limited to a small fraction of cells, or is it widespread? Quantitative data are required to clarify this point. Furthermore, does the degree of co-expression align with the scRNA-seq data? If there is a discrepancy, please discuss the potential causes. On a related note, SPCH expression has been analyzed in previous studies; why was this late-stage expression overlooked in the past?

This is a good point. While the images in the main figures are specifically of cells that express both SPCH-mCit and FAMA-mTurq2, we have now acquired additional images of the SPCH/FAMA double reporter to quantify this in an unbiased manner (and using sequential scanning to avoid bleed- through). Using these, we find that 2.1% of 2550 cells across 17 independent leaves that express *FAMA* also express *SPCH* in cotyledons 4 days after germination (0-7.2% across leaves). The overlap is limited to late guard mother cells (GMCs) and early guard cells (GCs) with SPCH levels decreasing quickly during GC development. We never detected SPCH in fully mature GCs, after pore formation. Overall, this is consistent with 2 out of 3 published scRNA-seq studies (3-28%) and, while this is a low fraction, it is important to consider co-expression only occurs in young GCs and GMCs while most of the *FAMA*-expressing cells are mature GCs that no longer express *SPCH*. When excluding the stomatal complexes with a large pore, the percentage of cells co-expressing *SPCH* and *FAMA* increases to 5.37%. We have included this additional data in Figure 1D. The percentage of cells where both fluorescent reporters can be observed is still overall lower than that observed in scRNA- seq datasets. Differences could be explained by 3 factors. First, detection limits differ significantly between transcripts and fluorescently tagged proteins. Secondly, stability of the SPCH protein is heavily regulated by EPIDERMAL PATTERNING FACTOR (EPF)-ERECTA (ER) signaling, which directly influences accumulation of SPCH protein but not its transcripts. Finally, the percentage of cells where overlap between SPCH and FAMA is detected depends heavily on what cells are counted. We counted all FAMA expressing cells which includes many mature GCs. Different scRNAseq datasets likely collected different proportions of mature GCs, influencing the % of FAMA+ cells expressing SPCH. Overall we believe these additional quantifications confirm and clarify how many cells have both SPCH and FAMA, in accordance with previously published data.

As the reviewer mentions, SPCH presence in the later stages of the stomatal lineage was previously largely overlooked. However, this is not entirely the case: in the original publication on SPCH (MacAlister 2007), the *SPCH* promoter was shown to be active in some GCs. We attribute the lack of focus on SPCH in GCs in part to the relatively lower levels of SPCH protein at this stage. A larger part we attribute to the reporters used. Until Lopez-Anido et al. 2021, all published translational reporters used the cds fragment for SPCH. However, it appears that the inclusion of introns, particularly the first intron, alters expression and protein presence.

3. Lines 255-267: The rationale for altering the DEG analysis method based solely on GO analysis results, without apparent scrutiny of the specific gene lists, is unclear. Additionally, the description of the subsequent analysis is too brief in the Results section compared to the explanation of why the change was made. Please provide a more detailed explanation of the analytical process. Did the authors include data for DEX induction at 0 hours as a control?

We agree with the reviewer that the rationale for modifying the bioinformatic analysis was not clear enough in the text and have adjusted this in the manuscript. In addition, we further outline our rationale for the interpretation of the GO analysis and the subsequent changes made in the approach below.

We first compared our FAMAp>>SPCH and FAMAp>>FAMA samples to the FAMAp>>NLS control directly. At first glance, the GO analysis revealed that genes downregulated upon both SPCH and FAMA induction were enriched in terms related to cell division. We found this particularly striking given that SPCH promotes asymmetric divisions. Looking more closely into the genes underlying this GO enrichment, we indeed found a consistent down-regulation of cell division genes that did not match the observed phenotypes. However, we did not make a decision to alter the analysis based on this alone. Instead, we inspected more carefully the fluorescence activated cell sorting (FACS) approach used for sample collection. We reasoned that the much brighter signal in the

© 2026. Published by The Company of Biologists under the terms of the Creative Commons Attribution License (https://creativecommons.org/licenses/by/4.0/).

NLS sample likely led to those samples containing more guard mother cells (GMCs) which would otherwise be too dim in other samples. We believe that this shift in cell type composition underlies, at least partly, the unexpected GO enrichment results. To correct for this bias, we decided to instead compare samples over time. Because this was not part of our original plan, we do not have samples at 0 hours. The fluorescent tag used for FACS is fused to the transcription factor of interest to monitor induction but, as a result, fluorescence is absent before induction and collection at 0h would have been impossible. Indeed, if we had been able to anticipate this issue we would have designed our approach differently, but at the time we preferred a more direct readout of the induction. Instead, for each transgenic (i.e., FAMAp>>FAMA and FAMAp>>SPCH), we performed three pairwise comparisons over time: (1) 2h vs. 3h, (2) 4h vs. 3h and (3) 4h vs. 2h. In the interest of identifying genes differently regulated by SPCH and FAMA, we additionally performed pairwise comparisons between FAMA and SPCH samples for each time point. In total, these six statistical tests identified 1,552 unique differentially expressed genes (DGEs) which we used as a starting point for the selection of candidate targets.

Regarding the final selection of target genes, we have now expanded and provided more details in both the Methods and Results sections. We now more clearly outline the selection steps, how the considered published datasets were used and what criteria were used for the final target selection. In short, we initially used available ChIP-seq and scRNAseq data to select genes that could be bound by SPCH or FAMA and were expressed during stomatal development. Finally, we selected genes with either (1) predicted functions that we believed might impact GC division or morphology, or (2) expression dynamics in the late lineage indicating strong or sudden up- or downregulation.

4. Fig. 2: Quantitative results are necessary for this figure. Currently, only a few representative images are presented, making it difficult to assess the frequency of the observed phenomena. Providing quantitative data would significantly enhance the reliability of these findings.

In this new version we have provided a quantitative and statistical analysis of these phenotypes. For the abnormal guard cell morphologies caused by SRDX-SPCH and SRDX-FAMA induction (in Fig. 2A-C), we have quantified the frequency of the major phenotypes that we observed and confirmed that these are more prevalent in transgenics than in wild-type leaves. We have included this quantification as Fig. 2D. For the phenotypic characterization of *fama* mutants complemented with FAMAp>>FAMA and FAMAp>>SRDX-FAMA (now in Fig. 2E-J), the supplementary figures already contained quantitative support which we have now decided to move to the main figure as panel Fig. 2L to support our claims.

5. It is known that the FAMA gene is expressed in myrosin cells in addition to guard cells. Is there a possibility that myrosin cells were included in the FAMA-positive population collected for expression analysis? At the very least, the authors should mention in the text that the FAMA promoter is also active in myrosin cells.

This is a good point and while we do not think this has a significant effect on our analysis, we agree it is good to mention in the results and expand on slightly in the discussion. We have added a sentence in the results (line 271-272) and a short fragment in the study limitations (line 580-581).

Minor points

6. Line 32: The background description in the Abstract is too broad. Focusing on specific issues relevant to this study would improve readability.

We agree with the reviewer and have shortened and rephrased the first half of the abstract. We now believe the focus and findings from this study are more obvious and accessible.

7. Line 54: Is the keyword "FMA" necessary?

Good point, we have removed it.

© 2026. Published by The Company of Biologists under the terms of the Creative Commons Attribution License (https://creativecommons.org/licenses/by/4.0/).

8. Line 137: Should the citation be "Zhang et al., 2021"?

True, we have updated the citation.

9. Line 143: Similar to Fig. 1, Fig. S2 requires calibration bars for fluorescence intensity and legends for the pseudocolors.

We have included a calibration bar for fluorescence intensity in Fig. S2.

10. Line 210: The sentence starts with "Interestingly," but is the context clear to readers without a reference? Citing a study such as (Ohashi-Ito and Bergmann, 2006) might help explain why this result is notable.

Thank you for this comment. We agree and have expanded this sentence to include more background on FAMA's activity and the implications of this result.

11. Fig. 2: Please specify which panels correspond to the magnified views in C and J.

Thank you. We have modified the figure legend to better reflect what the magnified views correspond to. In short, these are just zoomed-in photographs to better capture the types of phenotypes that we observed in the figures immediately above these two panels.

12. Fig. 2: The figure legend for panel C is duplicated.

We have fixed this now.

13. Fig. S6: The figure panels and the legend do not match (e.g., there is no legend for panel F). What does "17%<10" in panel F represent? Please also explain "total, long, trans" in panel J. Does the number "8" indicate the sample size (n)?

Thank you, we have now carefully reviewed all of these points and adjusted the figure and its legend for clarity. The % indicates the percentage of leaves showing less than 10 stomatal complexes with pores, indicating a *fama* phenotype: we have now clarified this in the figure legend.

14. Lines 223-225: It is unclear whether the authors are discussing the effects of ectopic overexpression or endogenous SPCH function. Please clarify.

We have altered this sentence with the main change being the inclusion of “can” to better reflect the uncertainty of whether SPCH always regulates these genes at this stage.

15. Line 275: Should the reference be "Figures 3F-I" instead of "3F-G"?

Thank you, indeed the reference was mistaken here, we have now updated the figure panels and reviewed all figure callouts.

16. Fig. S8A / Table S2: Since GLV1 is shown in Fig. S8A, it should also be listed in Table S2.

Indeed, thanks for noticing this. GLV1 is a synonym of RGF6, which we had already included in the Table S2. We have now used the RGF6 gene symbol in the figure for consistency.

17. Fig. 4: Panels D and L are both labeled "C". In panel L, the data points for "FAMAp::WSB opening" are solid dots, whereas others are open circles; please ensure consistency.

Thank you for pointing this out, we have fixed it now.

18. Lines 372-374: The text description does not match the cited data in Fig. 5E (Nucleus area). The correct citation appears to be Fig. S11G-I.

We have fixed this now.

© 2026. Published by The Company of Biologists under the terms of the Creative Commons Attribution License (https://creativecommons.org/licenses/by/4.0/).

19. Fig. 5F: I was unable to locate the reference to Fig. 5F in the main text. Please check if it is cited.

Thanks for catching this, we have now referenced the summary diagram (Fig. 5F) where it is relevant throughout the Discussion and Conclusion.

Reviewer 2
This manuscript presents a compelling and timely investigation into the regulatory mechanisms controlling the late stages of stomatal development. The study effectively challenges the classical, linear model of bHLH transcription factor action by demonstrating that SPCH and FAMA are co-expressed and functionally interact in GMCs and young GCs. The combination of precise genetic manipulations, cell type-specific transcriptomics, and detailed phenotypic characterization provides strong evidence for a model where the relative levels and activities of SPCH and FAMA fine-tune the final steps of stomatal formation. Here are the major concerns:

1. The data strongly suggest that SPCH and FAMA compete for binding to overlapping genomic targets. However, this competition model is primarily inferred from the similar phenotypes of the SRDX fusions and the rescue experiments in fama. More direct evidence is needed. Is there direct interaction of FAMA with SPCH? Do they enrich on the same promoter regions of their common target genes?

We agree that none of our experiments provide direct experimental evidence for competition between SPCH and FAMA. To answer your questions:

- Comparison of their ChIPseq peak suggests that most peaks are shared (55% of SPCH peaks, 64% of FAMA peaks), indicating that both transcription factors share a large fraction of their binding sites (Liu and Mair et al. PLOS Biology 2024).
- Interaction between SPCH and FAMA has not been shown. SPCH was not found in proximity labeling experiments using FAMA as bait (Mair et al. eLife 2019). While we attempted to confirm the interaction using FRET-FLIM experiments in the leaf, the low levels of SPCH prevented us from getting reliable results: SPCH levels were too low to perform lifetime measurements while for FAMA we only expect a small fraction of the protein to engage in such an interaction.
- In addition, in a model where SPCH and FAMA compete for the binding of target promoters, direct interaction between the two may not be required. There, we would expect competitive binding. However, we believe that the experiments to test this are outside of the scope of this paper.

While we currently mention the hypothesis that SPCH and FAMA compete in the text, we do not claim to have proven that this is how SPCH acts in the late lineage. Instead, we focused on understanding how their targets act in guard cell development. To avoid confusion, we have now explicitly stated that evidence for competitive binding or interaction is missing in the Results section. In addition, we have added how the mechanism for their interaction/competition is still unclear in the Discussion.

2. The results are descriptive in several key areas. For instance, the statement that SPCH-induced divisions are "mostly longitudinal" (Figure 1) requires rigorous quantification across multiple samples and time points. The percentages of different division types (longitudinal, transverse, aborted) should be quantified and statistically compared.

Thank you for your feedback. We have now performed quantitative analyses of our images, particularly for observations in Figures 1 and 2. For Fig. 1, we have now quantified the percentage of cells that co-express *SPCH* and *FAMA* in our double translational reporters (now Fig. 1D). In our FAMAp>>SPCH line, where *SPCH* is misexpressed in the late stomatal lineage, we previously reported observations of abnormal cell divisions, mostly longitudinal. We have now quantified the frequency of these additional divisions and confirmed that this is true at different developmental timepoints, i.e., 5 days after germination (dag), 11 dag and 16 dag. We believe that these data, which we have now included in Fig. 1H, provide more robust evidence for our claims. To further support these findings we also varied the timing of induction, contrasting

© 2026. Published by The Company of Biologists under the terms of the Creative Commons Attribution License (https://creativecommons.org/licenses/by/4.0/).

constant growth on DEX with seedlings that experienced 24 hours of 30uM DEX 4 days after germination after which they were again grown without DEX. As expected, this resulted in fewer complexes with additional divisions compared to constant growth on DEX. These are now included as a supplementary figure and referenced in the text. In Fig. 2, we have also performed additional quantitative analysis of abnormal guard cell phenotypes caused by misexpression of dominant negative SRDX-SPCH and SRDX-FAMA (now in Fig. 2D), which supports the observations from Fig. 2A-C. For clarity, we have also moved quantitative graphs from the supplementary figures to the main panels, which support phenotypes observed for *fama* mutants transformed with FAMAp>>FAMA and FAMAp>>SRDX-FAMA in Fig. 2D-J.

3. The finding that SRDX-FAMA can prevent tumor formation in the fama mutant but not rescue differentiation is fascinating. The authors interpret this as FAMA's differentiation function requiring its activator role. However, an alternative explanation is that the SRDX domain simply renders the protein non-functional, and the prevention of tumors is an artifact (e.g., by sequestering essential co-activators needed for division).

We thank the reviewer for the suggestion. Like any fusion, the SRDX domain might indeed render the FAMA protein non-functional. However, in that case we would not have expected it to rescue any aspects of the mutant phenotype and the fact that it prevents the formation of additional GC divisions suggests that it is at least functional in this aspect. As far as we are aware, FAMA's prevention of GC division relies on active gene repression rather than co-activator sequestration. However, it is difficult to untangle whether the reduced function is the result of non-functionality or of the SRDX domain removing the ability of FAMA to activate GC maturation genes. We have included it in the discussion (line 554-555) to inform the reader that this could be a possibility.

4. To determine the target genes of FAMA and SPCH in stomatal development, the authors used stomatal density, however, the density is often influenced by the size of pavement cells, therefore, the stomatal index is required to support their function.

We agree that stomatal density is influenced by pavement cell size and that stomatal index would avoid this confounding effect. However, in these experiments, we expressed genes of interest late in the stomatal lineage (using the *FAMA* promoter) and not in pavement cells or pavement cell precursors. Thus, we believe that pavement cell size or specification is unlikely to be affected by our genetic manipulations and that our observations of affected stomatal densities are a result of genetic defects in the stomatal lineage specifically. More importantly, we only use stomatal density as an initial selection step to select genes for further analysis. After this, we do not discuss the effects of any of these genes on early stomatal development or the final density/index in the context of broader leaf development. In this new version of the manuscript, we have clarified the purpose of measuring stomatal density as an initial readout for our target genes of interest (line 347-348).

5. In Figures 4C and S11, the authors measured the distance between guard cell membranes. It is important to note that plant cells possess a cell wall; therefore, are these measurements indicative of pore apertures?

Indeed pore aperture is determined by both guard cell deformation and by cell wall properties and the distance between membranes does not equal the opening that is formed. We have adjusted the text describing our experiments (line 369-371) as well as the discussion (line 523-524).

However, we do believe these measurements are indicative of both guard cell behavior, which is what we aim to measure here, and our pore apertures. What we are testing is the ability of WSB OX Guard Cells to open since we observed little to no distance between the membranes of most GC complexes under normal conditions, indicating closed or nearly closed pores. The measurement of distance between membranes in response to treatments gives us a good indication of cell behavior and capacity. However, indeed the reduced distance between membranes might not reflect actual pore aperture if cell wall thickness is altered in this line as well. We do believe that our measurements, while not directly measuring pore aperture, do reflect changes in pore aperture since we find that thickness of cell wall and pore deposition

© 2026. Published by The Company of Biologists under the terms of the Creative Commons Attribution License (https://creativecommons.org/licenses/by/4.0/).

are not significantly affected upon WSB misexpression as observed in TEM images.

6. It was reported that SCAP1 has a broader expression pattern in stomatal lineage cells, therefore, it cannot be used as guard cell marker line.

We agree with the reviewer that previously published GUS reporters for SCAP1 show broader signal in true leaves (Negi et al 2013 Curr Biol; Castorina et al. 2016 BMC Plant Biology). However, this is not the case for cotyledons and both reports show GC-specific expression in cotyledons. With our reporters, we observe GC-specific expression in cotyledons, similar to other fluorescent reporters published the past year (Shirakawa et al 2025 Nat Plants).

We have confirmed this by collecting new images for the reporter in wild-type plants, as shown below. We only detected *SCAP1* expression in guard cells, and in no case we found fluorescence in earlier stomatal lineage cells or in other leaf cell types (e.g., vasculature or mesophyll), supporting the specificity of the line.

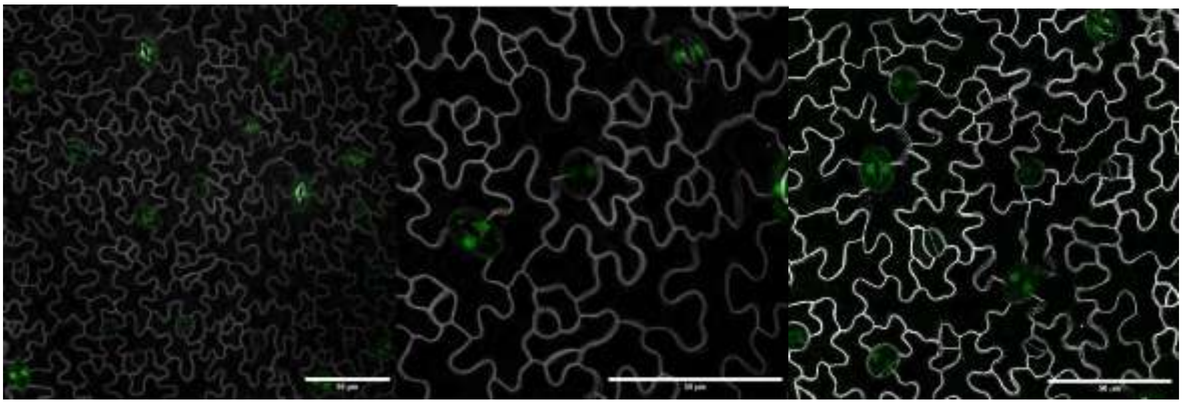

7. In most figure legends, the descriptions are not clearly articulated.

Thank you for this comment. We recognize that in several figures, especially in the supplement, critical information was missing. We have now gone over all figure legends and adjusted them to be more clearly articulated.

Reviewer 3

This manuscript addresses stomatal guard cell differentiation from the perspective of master transcription factor regulation. Building on a previously unexpected observation from the Bergmann lab that the transcription factor SPEECHLESS (SPCH) is expressed during late stages of the stomatal lineage (Lopez-Anido et al. Developmental Cell), the authors perform a thorough and well-designed analysis of SPCH and its sister transcription factor FAMA. In the first half of the paper, the authors compare inducible expression systems for SPCH and FAMA, as well as SRDX-fused chimeric repressor versions (SPCH-SRDX and FAMA-SRDX), to dissect functional differences between these transcription factors. In the second half, they carefully analyze a set of shared downstream genes of SPCH and FAMA using misexpression approaches, ultimately classifying their roles in guard cell division and expansion and successfully expanding the regulatory network. Overall, this study provides important molecular insight into how guard cell maturation and morphogenesis are regulated, and I strongly recommend this manuscript for publication in Development. I do not think additional experiments are strictly required. However, clarification of several points and the inclusion of additional figures for data that are currently only described in the text would substantially improve clarity and accessibility for readers.

Major comments

1. Discussion of SPCH-FAMA interaction at the protein level. It would benefit the reader to include a discussion of whether SPCH and FAMA physically interact at the protein level. In the FAMA-TurboID dataset reported by Mair et al. (eLife, 2019), SPCH was not detected among FAMA interactors. It may be useful to explicitly mention this in the Discussion and comment on how this result fits with the current findings.

© 2026. Published by The Company of Biologists under the terms of the Creative Commons Attribution License (https://creativecommons.org/licenses/by/4.0/).

See also comment 1 from Reviewer 2. To avoid confusion, we have now explicitly stated that evidence for competitive binding or interaction is missing in the Results section. In addition, we have added how the mechanism for their interaction/competition in the regulation of target genes is still unclear in the Discussion.

2. Definition and validation of "transcriptional targets" in the title and Results. The title refers to "transcriptional targets of SPEECHLESS and FAMA," but it is not always clear whether the genes analyzed in the second half of the manuscript are direct targets, or whether they are positively or negatively regulated by SPCH and/or FAMA.

We agree that, while we aimed to find direct transcriptional targets by using short induction times and cross-checking with published ChIP-seq data, we cannot unequivocally claim that these are direct targets. We have now changed the manuscript title to “Targets of SPEECHLESS and FAMA control guard cell division and expansion in the late stomatal lineage” and refer to the genes in question as "putative targets” to reduce this implication.

   a. Clarification of target selection strategy. Although I read the Methods section carefully, the logic used to narrow down candidates was not entirely clear. Specifically, it was difficult to understand whether the authors focused primarily on genes already known to be involved in stomatal maturation, or whether the candidate genes were selected purely based on intersection of multiple omics datasets. It would be very helpful if the authors could clarify this point explicitly. For example, a schematic or Venn diagram illustrating how the candidate list was reduced (e.g. from ~195 genes to 23 genes) would greatly improve clarity.

We agree with the reviewer that the rationale for the selection was not clear enough in the text and have adjusted this explanation in both the Results and Methods sections.

In the Results section (lines 314-329), we now include the main selection steps and explain how we used the published datasets for the final selection of candidate targets. In short, we started from a total of 1,552 differentially expressed genes (DEGs) significantly affected by induction of SPCH and/or FAMA in our FAMAp>>SPCH and FAMAp>>FAMA transgenics. First, we used available ChIP-seq data for SPCH (Lau et al. 2014) and FAMA (Liu et al. 2024) to select genes that could be bound by SPCH and/or FAMA. This narrowed down the list of candidates from 1,552 to 863. Next, we used scRNAseq data (Lopez-Anido et al. 2021) to select genes expressed in the stomatal lineage. To this end, we manually scored expression patterns of the 863 genes and selected those assigned with the following categories:

1. “Young guard cell”, i.e., genes preferentially expressed in guard cells soon after completion of the symmetric division.
2. “Young other”, i.e., genes expressed in early stages of stomatal and pavement cell lineages.
3. “Old guard cell”, i.e., genes preferentially expressed in more mature guard cells.
4. “Stomatal”, i.e., genes expressed broadly throughout the stomatal lineage but not in pavement cells.
5. “Differentiating”, i.e., genes preferentially expressed in differentiating pavement cells and guard cells.
6. “Early stomatal”, i.e., genes preferentially expressed in stomatal lineage precursors.

Selecting only genes with either of these 6 categories further narrowed down the list of candidates from 863 to 265. Finally, from the 265 remaining genes, we manually selected 23 genes with either predicted functions that we believed might impact GC division or morphology, or (2) expression dynamics in the late lineage indicating strong or sudden up- or down-regulation. In this last step, we considered all information we had available, including previously published induction datasets (Lau et al. 2014, Lau et al. 2018, Hachez et al. 2011), detailed expression dynamics reported in scRNAseq data and predicted function as well as published results on each gene.

In the Methods (lines 725-750), we describe these steps in more detail as well as the

© 2026. Published by The Company of Biologists under the terms of the Creative Commons Attribution License (https://creativecommons.org/licenses/by/4.0/).

different categories/patterns of genes that we found among the final 23 candidates. For each gene, we have also included for which selection category it fell into in Supplementary Table S4. Regarding the schematic of the selection process, we have now updated the main steps in Figure 3. We followed the reviewer's suggestion to create a Venn Diagram (see below) but found that it did not add sufficient new information to include it in the Figure 3.

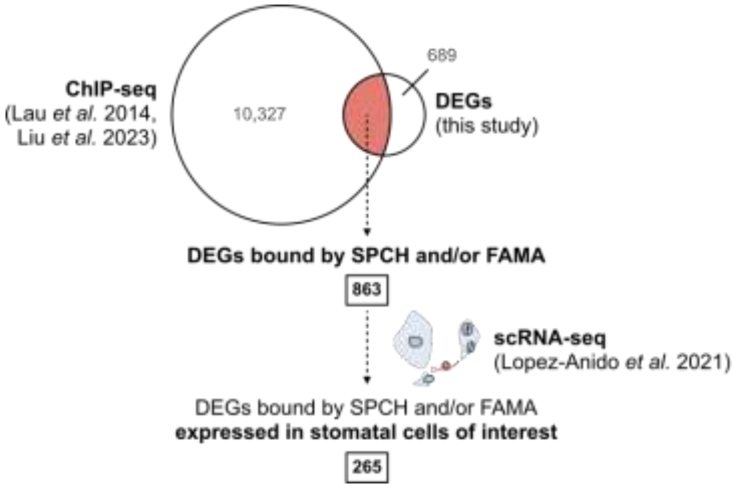

We have also considered a summary graph indicating how many candidate genes we selected for each selection category (see below), but do not believe this to be informative enough to add to the manuscript as we included a similar number of genes per class.

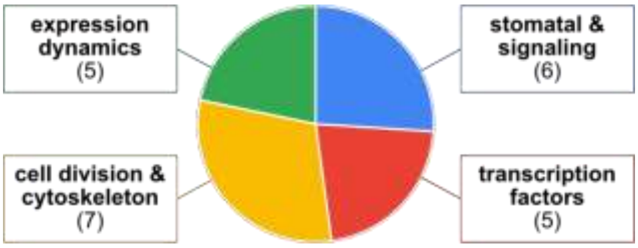

b. Inclusion of omics data in the main figures. In addition, I strongly recommend that representative omics data supporting the analyzed gene set be shown in the main figures rather than only being described in the text. Specifically, the following would be very helpful: (i) Screenshots of ChIP-seq peaks in IGV for representative target genes, (ii) Expression plots (mock vs. dex) for FAMA, SPCH, FAMA-SRDX, and SPCH- SRDX induction lines, (iii) Expression patterns of these genes projected onto the UMAP from the single-cell analysis by Lopez-Anido et al. (Developmental Cell). These additions would substantially strengthen the link between the transcriptomic analyses and the functional experiments.

We thank the reviewer for this suggestion. To strengthen the connection between our transcriptomic data and the subsequent experiments we have included the suggested plots for 4 genes in Figure 3. Due to space constraints, equivalent plots for the remaining genes that we discuss in more detail in the last sections of the manuscript are included in Supplemental Figure S9. As suggested, we have included for each gene: (i) diagrams of ChIP-seq peaks, (ii) expression plots in the stomatal lineage as detected by the single-cell transcriptome analysis by Lopez-Anido et al., 2021; and (iii) expression plots showing transcriptional changes upon FAMA and SPCH induction as detected in our own experiment.

<u>Minor comments</u>

- Line 44: The wording may give the impression that a physical interaction between SPCH and FAMA was directly tested; this could be clarified.

© 2026. Published by The Company of Biologists under the terms of the Creative Commons Attribution License (https://creativecommons.org/licenses/by/4.0/).

We have reworded this sentence slightly to avoid confusion.

- Line 68: The correct reference may be Ohashi-Ito and Bergmann, 2006.

This is true, we have now corrected the reference.

- Line 394 / Figure 5F: Consider adding SCAP1 to the model diagram.

We thank the reviewer for the suggestion and agree that it would make the diagram a better summary of guard cell development. However, we feel that, with that goal, we would need to add many additional genes. Instead, we focus on having this diagram as a summary of the results presented in this work and we now emphasize that more in the figure legend.

- Line 484: In the myrosin cell lineage, WSB represses SCAP1, whereas this does not appear to occur in the stomatal lineage. This contrast is very interesting and may deserve further discussion.

Thank you for this comment, we have now included this observation explicitly in the Discussion.

Second decision letter

MS ID#: dev.205374R1

MS TITLE: Targets of SPEECHLESS and FAMA control guard cell division and expansion in the late stomatal lineage

AUTHORS: Pablo González-Suárez, Yoëlle Hilbers, Alanta Budrys, Ao Liu, Sandra Richter, Dominique C. Bergmann and Margot Smit

Dear Dr Smit,

You will be pleased to see that the reviewers are happy with your revisions and that there are just a few minor points to address before proceeding to publication.

Reviewer 1

Advance summary and potential significance to field

I would like to thank the authors for their thorough and sincere response to my previous comments. The revised manuscript has been significantly improved through the addition of new quantitative data and the supplemental functional analysis using SPCH amiRNA lines. These additions appropriately address my major concerns regarding data reliability and the integration of these findings with established models.

I am satisfied with the current version of the manuscript and believe it provides important insights into the late-stage regulation of the stomatal lineage. However, I have a few remaining minor points regarding text clarity that I would encourage the authors to address to ensure the findings are as accessible as possible to a broad readership.

Minor Points regarding Content and Clarity:
Line 200: Regarding the sentence 'FAMA induction did not (Figure S5)', the intended meaning of 'did not' is ambiguous. It is unclear whether it refers to the preceding 'did not affect GC division...' or to the idea expressed in the Figure S5 title, 'does not abolish SPCH induction phenotypes.' I

© 2026. Published by The Company of Biologists under the terms of the Creative Commons Attribution License (https://creativecommons.org/licenses/by/4.0/).

recommend completing the sentence without ellipsis to accurately reflect the data in Figure S5 and avoid reader confusion.

Line 215: I appreciate the authors' effort to address my previous comment by expanding the description of FAMA's activity. The findings regarding FAMA-SRDX are indeed very interesting; however, I found the current sentence structure still somewhat difficult to parse. To ensure this key point is accessible to a broad readership, I would encourage a further revision to more clearly highlight the contrast between FAMA's typical role as an activator and the paradoxical observation that its repressive form (SRDX) effectively blocks cell division.

Line 359: Regarding the sentence 'Our expectation was that changes in stomatal...', I found the structure somewhat convoluted and difficult to follow. I suggest rephrasing or splitting this sentence to more clearly state how the inverse relationship between stomatal density and size served as a rationale for your selection criteria. This will help readers more easily grasp the logic behind your experimental design.

Additional Technical Corrections:
Line 179: There is an extra space after "N=17." Please remove it.

Line 183: The reference to "G-G'" should be corrected to "G." Panel G' does not exist in the figure and appears to be a leftover from a previous version.

Line 365: There is an extra space in "of XYL1." Please remove it.

Line 401 (Figure 4c and 4d): There is an inconsistent mix of uppercase and lowercase letters used to indicate statistical significance within the same graph. Unless this distinction has a specific scientific meaning, please unify the casing for clarity.

Line 429: The reference to "Figure S11G-I" appears to be an error and should likely be "Figure S13G-I." Furthermore, while the authors describe these panels as showing single guard cells, they are morphologically indistinguishable from regular two-celled stomata in these TEM micrographs. Please clarify the criteria or evidence used to identify these specific cells as single guard cells.

Line 531: There is an extra space in "landscapes and." Please remove it.

Line 609: Please use lowercase for "myrosin cells" (currently "Myrosin Cells") to follow standard biological nomenclature.

<u>Reviewer 2</u>

Advance summary and potential significance to field

Thank the authors for your reply and revisions. Most of the comments have been addressed, but I still have one issue that I feel needs to be resolved, which is the abstract. The current abstract does not clearly state the study's key findings and broader significance, and it includes an excessive amount of background information.

<u>Reviewer 3</u>

Advance summary and potential significance to field

The revised manuscript has adequately addressed my comments and is now of high quality. I have no further concerns and recommend acceptance.

© 2026. Published by The Company of Biologists under the terms of the Creative Commons Attribution License (https://creativecommons.org/licenses/by/4.0/).

Second revision

Author response to reviewers' comments

Reviewer 1: I would like to thank the authors for their thorough and sincere response to my previous comments. The revised manuscript has been significantly improved through the addition of new quantitative data and the supplemental functional analysis using SPCH amiRNA lines. These additions appropriately address my major concerns regarding data reliability and the integration of these findings with established models.

I am satisfied with the current version of the manuscript and believe it provides important insights into the late-stage regulation of the stomatal lineage. However, I have a few remaining minor points regarding text clarity that I would encourage the authors to address to ensure the findings are as accessible as possible to a broad readership.

Minor Points regarding Content and Clarity:

Line 200: Regarding the sentence 'FAMA induction did not (Figure S5)', the intended meaning of 'did not' is ambiguous. It is unclear whether it refers to the preceding 'did not affect GC division...' or to the idea expressed in the Figure S5 title, 'does not abolish SPCH induction phenotypes.' I recommend completing the sentence without ellipsis to accurately reflect the data in Figure S5 and avoid reader confusion.

We agree with the reviewer and have rephrased the sentence to improve clarity.

Line 215: I appreciate the authors' effort to address my previous comment by expanding the description of FAMA's activity. The findings regarding FAMA-SRDX are indeed very interesting; however, I found the current sentence structure still somewhat difficult to parse. To ensure this key point is accessible to a broad readership, I would encourage a further revision to more clearly highlight the contrast between FAMA's typical role as an activator and the paradoxical observation that its repressive form (SRDX) effectively blocks cell division.

In line 218-221 we have now more explicitly named FAMA’s role as a positive regulator of many of its targets as well as adding its known negative regulation of CDKB1;1. This more clearly highlights how the SRDX-FAMA findings fit or do not fit with FAMA’s known roles.

Line 359: Regarding the sentence 'Our expectation was that changes in stomatal...', I found the structure somewhat convoluted and difficult to follow. I suggest rephrasing or splitting this sentence to more clearly state how the inverse relationship between stomatal density and size served as a rationale for your selection criteria. This will help readers more easily grasp the logic behind your experimental design.

In this new version we have rephrased lines 362-366 into shorter, clearer sentences according to the reviewer’s recommendation.

Additional Technical Corrections:

Line 179: There is an extra space after "N=17." Please remove it.

We have removed this now.

Line 183: The reference to "G-G'" should be corrected to "G." Panel G' does not exist in the figure and appears to be a leftover from a previous version.

This has now been corrected.

Line 365: There is an extra space in "of XYL1." Please remove it.

We have removed this now.

© 2026. Published by The Company of Biologists under the terms of the Creative Commons Attribution License (https://creativecommons.org/licenses/by/4.0/).

Line 401 (Figure 4c and 4d): There is an inconsistent mix of uppercase and lowercase letters used to indicate statistical significance within the same graph. Unless this distinction has a specific scientific meaning, please unify the casing for clarity.

We have updated the figure to now have only small, regular font letters to indicate statistical significance.

Line 429: The reference to "Figure S11G-I" appears to be an error and should likely be "Figure S13G-I." Furthermore, while the authors describe these panels as showing single guard cells, they are morphologically indistinguishable from regular two-celled stomata in these TEM micrographs. Please clarify the criteria or evidence used to identify these specific cells as single guard cells.

We thank the reviewer for pointing out this error and have removed the sentence.

Line 531: There is an extra space in "landscapes and." Please remove it.

We have removed this now.

Line 609: Please use lowercase for "myrosin cells" (currently "Myrosin Cells") to follow standard biological nomenclature.

We have updated this to lowercase letters.

Reviewer 2: Thank the authors for your reply and revisions. Most of the comments have been addressed, but I still have one issue that I feel needs to be resolved, which is the abstract. The current abstract does not clearly state the study's key findings and broader significance, and it includes an excessive amount of background information.

We thank the reviewer for their comments and agree that the previous abstract did not clearly reflect all our findings and their broader significance. We have now largely rewritten the abstract and now use more than half to outline our findings and the broader significance.

Reviewer 3: The revised manuscript has adequately addressed my comments and is now of high quality. I have no further concerns and recommend acceptance.

Thank you for your comments.

Third decision letter

MS ID#: dev.205374R2

MS TITLE: Targets of SPEECHLESS and FAMA control guard cell division and expansion in the late stomatal lineage

AUTHORS: Pablo González-Suárez, Yoëlle Hilbers, Alanta Budrys, Ao Liu, Sandra Richter, Dominique C. Bergmann and Margot Smit

Dear Margot,

I am happy to tell you that your manuscript has been accepted for publication in Development, pending our standard publication integrity checks.

© 2026. Published by The Company of Biologists under the terms of the Creative Commons Attribution License (https://creativecommons.org/licenses/by/4.0/).
